# Systemic lupus erythematosus in pregnancy: Maternal and fetal outcomes and management strategies (Review)

**DOI:** 10.3892/mi.2026.319

**Published:** 2026-05-06

**Authors:** Isra Omar, Ahmed Alakhras, Mona Ahmad, Samahir Mutwali

**Affiliations:** 1 Department of Clinical Medicine, College of Medicine, Almaarefa University, Riyadh 11597, Kingdom of Saudi Arabia; 2 Department of Internal Medicine, College of Medicine, University of Medical Sciences and Technology, Kigali 250, Rwanda; 3 Royal College of Physicians of Ireland, Dublin 2, D02 E434, Ireland; 4 TeleGeriatric Michigan State University Fellowship Program, Michigan State University, East Lansing, MI 48824, USA

**Keywords:** systemic lupus erythematosus, pregnancy, maternal outcomes, fetal outcomes, lupus nephritis, antiphospholipid syndrome, preeclampsia, neonatal lupus, immunosuppressive therapy, high-risk pregnancy

## Abstract

Systemic lupus erythematosus (SLE) is a chronic, multisystem, autoimmune disease that predominantly affects women during their reproductive years. Pregnancy in women with SLE is no longer considered uniformly prohibitive; however, it remains a high-risk clinical scenario due to maternal disease activity, lupus nephritis, antiphospholipid antibodies, chronic organ damage, and treatment-related factors that can adversely affect both maternal and fetal outcomes. Current evidence indicates that the risk of flare is highest in women who conceive with an active disease, discontinue effective therapy, or have a history of major organ involvement. At the same time, pregnancy introduces diagnostic complexity due to physiological gestational changes which may mimic lupus activity, particularly in hematological, renal and vascular domains. Maternal complications include preeclampsia, thromboembolism, infection, lupus nephritis exacerbation and postpartum flare, whereas fetal complications include miscarriage, fetal growth restriction, prematurity, neonatal lupus and congenital heart block in anti-Ro/SSA- or anti-La/SSB-positive pregnancies. Optimal management begins prior to conception and requires multidisciplinary care, risk stratification, medication optimization and structured maternal-fetal surveillance throughout pregnancy and the puerperium. The present narrative review summarizes contemporary evidence on the immunopathogenesis, preconception assessment, maternal and fetal risks, and therapeutic management of SLE in pregnancy, with an emphasis on clinically applicable strategies that improve patient outcomzes.

## 1. Introduction

Systemic lupus erythematosus (SLE) is a chronic, autoimmune, connective tissue disease characterized by the loss of immune tolerance, autoantibody production, immune-complex deposition and multisystem inflammatory injury. It affects women disproportionately and is most frequently diagnosed during the reproductive years, thus necessitating pregnancy counseling and pregnancy management integral components of longitudinal lupus care (1-3). Although pregnancy outcomes in SLE have improved substantially over the past two decades, affected pregnancies still carry higher rates of maternal morbidity, placental dysfunction and adverse neonatal outcomes compared with pregnancies in the general population, particularly in the presence of active disease, lupus nephritis and antiphospholipid antibodies (4-6). Accordingly, the modern approach to SLE in pregnancy extends beyond flare management alone and requires preconception optimization, individualized risk assessment, pregnancy-compatible disease control, and coordinated surveillance by rheumatology, maternal-fetal medicine, nephrology and neonatology teams when indicated (7-9).

## 2. Pathogenesis of SLE

SLE develops through multilevel interactions among genetic susceptibility, epigenetic dysregulation, sex-hormone signaling, defective clearance of apoptotic debris, type I interferon activation, aberrant B-cell and T-cell responses and complement-mediated tissue injury. In genetically predisposed individuals, environmental triggers, such as ultraviolet radiation, viral exposure, cigarette smoke, and selected drugs increase cellular stress and apoptosis. When apoptotic material is inadequately cleared, nuclear antigens remain accessible to antigen-presenting cells and autoreactive lymphocytes, facilitating the generation of antinuclear, anti-double stranded DNA, anti-Sm and antiphospholipid antibodies. The resulting immune complexes activate complement, amplify plasmacytoid dendritic cell interferon signaling, recruit inflammatory leukocytes, and produce end-organ injury in the kidneys, skin, joints, hematologic compartment, vasculature and central nervous system (10-13).

Pregnancy adds a distinct immunologic layer to this process. Successful gestation requires finely regulated maternal immune tolerance rather than generalized immunosuppression. Early implantation and placentation rely on controlled innate immune activation, decidual natural killer-cell remodeling, trophoblast-maternal crosstalk and subsequent expansion of regulatory T-cell pathways. In patients with SLE, these tolerance mechanisms may be destabilized by pre-existing interferon pathway activation, complement overactivity, endothelial dysfunction, and persistent autoantibody production. A relative shift in T-cell polarization, dysregulated Th17/Treg balance, enhanced B-cell survival and heightened cytokine signaling may, in combination, increase flare susceptibility, particularly when pregnancy begins during a period of serologic or clinical activity (7,13-15).

**Figure 1:**
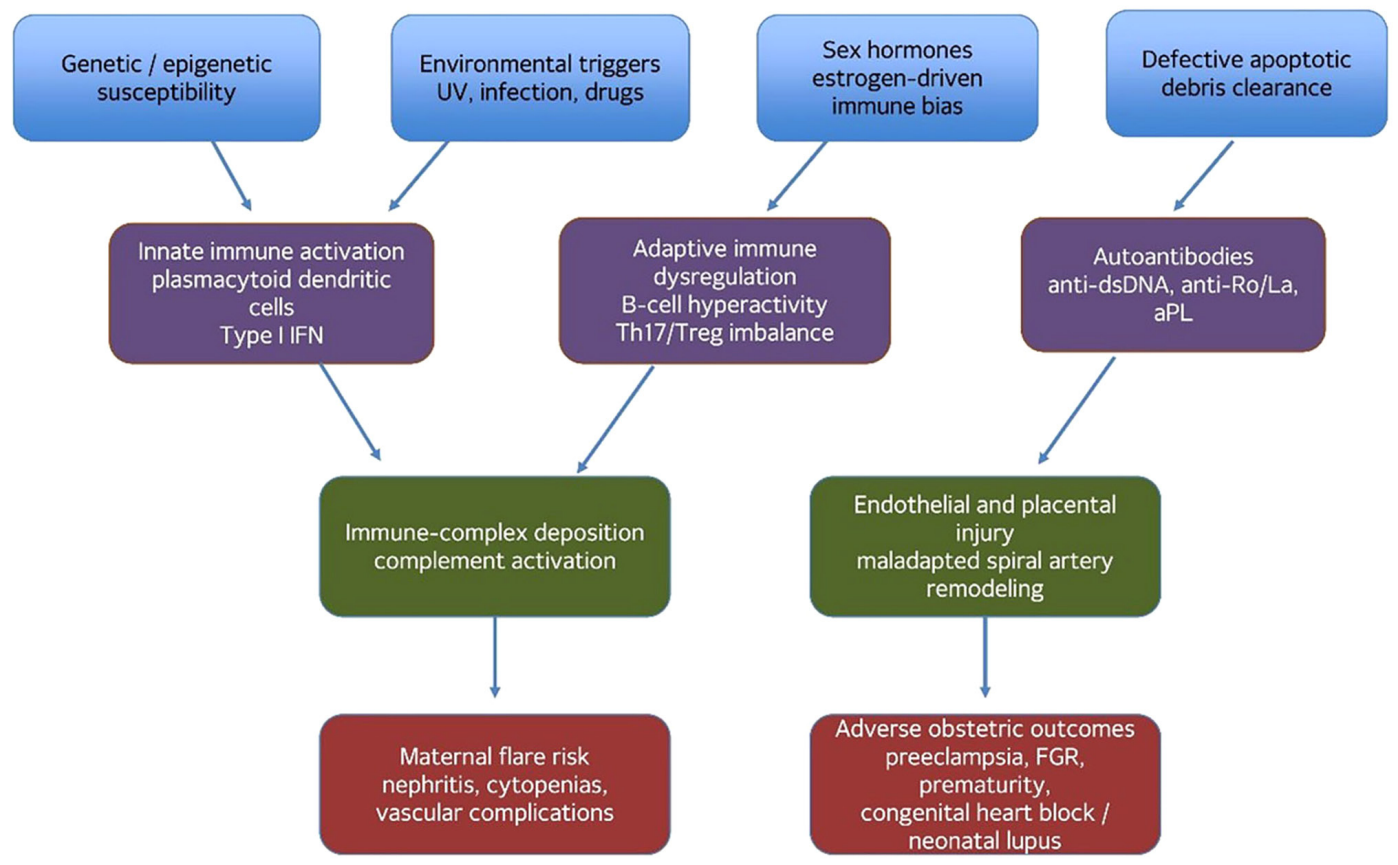
Schematic diagram summarizing the major immune and placental pathways linking systemic lupus erythematosus pathobiology with disease flare and adverse obstetric outcomes during pregnancy.

These mechanisms are also directly relevant to obstetric diseases. Type I interferon excess, antiphospholipid antibodies, neutrophil extracellular trap formation, complement activation, and placental vascular injury can impair spiral artery remodeling and uteroplacental perfusion, thereby linking maternal autoimmunity with preeclampsia, fetal growth restriction, preterm birth and fetal loss. Thus, the pathobiology of SLE in pregnancy should be understood not merely as baseline lupus occurring coincidentally during gestation, but as a dynamic interaction between systemic autoimmunity and the immunologic, vascular and placental adaptations required for normal pregnancy (4,5,9,13,15,16). The interaction between immune dysregulation, interferon signaling, complement activation and placental vascular injury provides a unifying framework linking systemic lupus erythematosus pathobiology with adverse obstetric outcomes. The schematic diagram presented in Fig. 1 highlights how immune-complex formation and endothelial dysfunction converge on placental insufficiency, thereby contributing to complications such as preeclampsia, fetal growth restriction and preterm birth.

## 3. Effects of pregnancy on SLE

The effects of pregnancy on SLE activity are best understood through the interplay between pre-pregnancy disease control, medication continuation, serologic activity and prior organ involvement rather than pregnancy alone. Contemporary cohorts suggest that flare rates are substantially lower than historically reported when conception occurs during remission or low disease activity and hydroxychloroquine is continued; nevertheless, pregnancy and the early postpartum period remain clinically vulnerable windows, particularly for patients with lupus nephritis, active serology, previous severe flares, or the withdrawal of pregnancy-compatible therapy (4-6,16-20).

The assessment of activity during pregnancy should be standardized whenever possible. The SLE Pregnancy Disease Activity Index (SLEPDAI) was specifically developed to reduce confounding by physiological gestational changes, and recent prospective evidence supports the utility of the SLE disease activity score (SLE-DAS) in early pregnancy as a predictor of subsequent maternal flares, particularly as demonstrated in first-trimester cohort analyses (18). In parallel, general disease activity instruments, such as SLEDAI, BILAG-2004 and Physician Global Assessment (PGA) remain useful when interpreted by clinicians experienced in lupus and pregnancy, while the SLICC/ACR Damage Index is relevant for defining cumulative organ injury and long-term maternal risk rather than active inflammation per se (8,18-22). From a practical standpoint, these instruments should not replace clinical judgment; however, they improve reproducibility, longitudinal monitoring and communication across specialties.

A major clinical goal is the achievement of remission or low disease activity prior to conception. A low lupus disease activity state (LLDAS) and remission at conception have been associated with fewer maternal flares and more favorable obstetric outcomes, while first-trimester disease activity and hypocomplementemia are associated with preterm birth, preeclampsia and placental insufficiency (18-20,22). Distinguishing the physiological changes of pregnancy from flare remains challenging: Mild anemia, edema, fatigue and isolated musculoskeletal discomfort may be gestational, whereas a falling complement level relative to baseline, rising anti-dsDNA titers, active urinary sediment, progressive proteinuria, thrombocytopenia, or inflammatory rash/arthritis should prompt concern for active disease. The postpartum period deserves equal attention as disease activity often intensifies following delivery, particularly within the first 6 months (16,19).

## 4. Pre-pregnancy counseling and considerations

Pre-pregnancy counseling should be structured, explicit and individualized as the strongest determinant of pregnancy outcome in SLE is not the diagnosis alone, but the disease state and treatment profile at conception. Counseling should therefore move beyond a general warning that ‘complications may occur’ and instead classify the patient into an obstetric-risk framework based on current disease activity, cumulative organ damage, antibody profile, renal history, cardiovascular status, prior obstetric history and exposure to potentially teratogenic agents (4,5,8,16,23).

The ideal candidate for conception is a patient whose disease has been clinically quiescent or maintained at a low disease activity for at least 6 months, with stable renal function, controlled blood pressure, pregnancy-compatible medications and no major recent flares. By contrast, conception during active nephritis, uncontrolled hypertension, pulmonary hypertension, advanced chronic kidney disease, severe heart failure, recent stroke, or active thrombotic antiphospholipid syndrome (APS) is associated with a substantially increased maternal and fetal risk, and may justify the postponement of pregnancy until medical optimization is achieved (5,7,8,16). Counseling should explicitly differentiate modifiable risk from non-modifiable risk: Disease control, medication reconciliation, aspirin/heparin planning and the timing of conception are modifiable; prior severe nephritis and chronic organ damage define baseline vulnerability.

Medication optimization is a central component of pre-pregnancy care. Hydroxychloroquine should generally be continued; prednisone should be minimized to the lowest effective dose; azathioprine, tacrolimus and cyclosporine may be considered when indicated; and known teratogens, such as mycophenolate, methotrexate, cyclophosphamide and leflunomide require discontinuation with appropriate washout and transition planning prior to conception (8,9,15,24). In addition, counseling should address aspirin prophylaxis, anticoagulation when APS is present, anti-Ro/SSA and anti-La/SSB-related fetal surveillance, and the need for multidisciplinary care. Notably, modern data underscore that pregnancy planning and medical readiness are associated with improved reproductive outcomes than ill-timed conception during active disease or teratogenic exposure (23). A structured preconception assessment framework integrating disease activity, organ involvement, antibody profile and medication status supports individualized risk stratification and informed decision-making regarding pregnancy timing (Table I).

## 5. Pre-pregnancy analyses in patients with SLE

The pre-pregnancy evaluation in SLE should distinguish between routine background lupus surveillance and pregnancy-specific risk stratification. General SLE monitoring includes complete blood count, renal profile, liver profile, urinalysis, protein quantification, complement levels and anti-dsDNA assessment; however, in the context of pregnancy planning, these tests have a different purpose: They establish a maternal baseline against which later gestational changes can be interpreted and identify women at increased risk for flare, nephritis recurrence, preeclampsia, placental dysfunction and fetal compromise (8,16,21).

Pregnancy-specific evaluations should include, at minimum, the confirmation of disease activity status; the quantification of proteinuria and renal function; blood pressure assessment; antiphospholipid antibody profiling; anti-Ro/SSA and anti-La/SSB testing; medication review; and the targeted evaluation of organ systems that materially alter pregnancy risk, particularly the kidneys, cardiovascular system and lungs. Echocardiography is appropriate when pulmonary hypertension, cardiomyopathy, significant valvular disease, or prior cardiopulmonary symptoms are suspected. Pulmonary function testing or thoracic imaging should be reserved for women with respiratory symptoms or known parenchymal disease rather than performed indiscriminately in all patients. Likewise, thrombophilia-oriented planning should focus primarily on APS rather than broad nonselective testing (5,7,8,16).

Baseline complement levels and anti-dsDNA titers are particularly valuable as serial interpretation during pregnancy is more informative than isolated absolute values. Similarly, documenting preconception serum creatinine, urine protein excretion and urinary sediment markedly improves later differentiation between quiescent renal disease, lupus nephritis flare and superimposed preeclampsia. For women with thyroid symptoms, prior thyroid disease, or autoimmune clustering, thyroid function testing is reasonable; however, thyroid studies should be presented as selective adjuncts rather than mandatory lupus-specific pregnancy biomarkers in all patients. This distinction improves conceptual clarity and aligns the evaluation with practical clinical decision-making (8,16,21). Establishing a comprehensive maternal baseline through targeted laboratory and clinical evaluation is essential for distinguishing physiological gestational changes from pathological processes such as lupus flare, nephritis, or preeclampsia (Table II).

## 6. Contraceptives in patients with SLE

Contraception is relevant in patients with SLE as it underpins safe pregnancy planning. Women with active disease, recent flares, severe organ involvement, or current exposure to teratogenic medications should be protected from unplanned conception until disease control and medication transition have been achieved. Accordingly, contraceptive counseling should be framed as a component of reproductive safety and timing rather than as a disconnected gynecologic topic (8,23).

Highly effective contraception is particularly important for women taking mycophenolate, methotrexate, cyclophosphamide, or other agents that may cause embryotoxicity or fetotoxicity. Intrauterine devices, including copper and levonorgestrel systems, are generally preferred due to their high efficacy and minimal dependence on adherence, although the thrombotic profile and antiphospholipid antibody status should be considered when estrogen-containing methods are discussed. Combined oral contraceptives may be acceptable in selected women with stable, low-activity disease and without antiphospholipid antibodies or a major risk of thrombosis, whereas progestin-only options are often preferred when thrombosis risk is a concern (8,16,25).

The essential clinical principle is that every woman of reproductive age with SLE should have a documented reproductive plan: Whether she desires pregnancy now, later, or not at all; whether conception is medically advisable at the present time; and which contraceptive strategy best aligns with her disease activity and medications. This approach prevents high-risk pregnancies during periods of medical instability and directly supports the goal of well-timed conception (23).

## 7. Infertility in patients with SLE

Fertility in women with SLE is often described as broadly preserved; yet, this statement may be misleading in clinical practice. Subfertility in SLE may arise from disease-related inflammation, chronic organ dysfunction, reduced ovarian reserve, associated autoimmune endocrinopathies, sexual dysfunction, psychosocial burden, delayed childbearing and treatment-related gonadotoxicity. Therefore, the infertility discussion should extend beyond the mere observation that infertility ‘is not uncommon’ and instead outline a diagnostic and management framework tailored to lupus (15,26-28).

The initial evaluation should mirror standard infertility work-up, but with SLE-specific expansion. Core assessment includes ovulatory history, ovarian reserve testing with anti-Mullerian hormone and/or antral follicle count, thyroid assessment when indicated, a review of menstrual function, age-related fertility risk, partner-related factors and medication exposure. Particular attention should be paid to cumulative cyclophosphamide exposure, as gonadal toxicity is dose- and age-dependent and remains one of the most critical iatrogenic threats to fertility in SLE. Recent data support the use of anti-Mullerian hormone and antral follicle count as clinically useful markers of diminished ovarian reserve in this population, even when menstrual cycles appear preserved (27). These markers are particularly useful because cyclophosphamide-related ovarian injury may precede overt menstrual disturbance, and contemporary reviews of fertility in SLE recommend incorporating ovarian reserve testing into individualized reproductive counseling (26,27).

Fertility preservation should be discussed prior to the e of cyclophosphamide whenever feasible. Concurrent gonadotropin-releasing hormone agonists may reduce the risk of ovarian insufficiency and improve the odds of later pregnancy, although they should be viewed as protective rather than fully restorative. When disease severity permits, mycophenolate-based nephritis regimens may reduce gonadotoxic exposure compared with cyclophosphamide; however, this advantage is relevant to long-term reproductive planning and not to pregnancy itself, as mycophenolate is contraindicated during gestation (28). Assisted reproductive technologies may be considered in carefully selected patients with quiescent disease; however, stimulation protocols should be coordinated with rheumatology and maternal-fetal medicine teams because estrogen exposure, thrombosis risk, and antiphospholipid antibodies may alter the safety profile. Anticoagulation and hydroxychloroquine continuation may be necessary in selected patients undergoing assisted reproduction (8,26,29).

## 8. Effects of SLE on pregnancy

The effects of SLE on pregnancy should be discussed in pregnancy-specific terms rather than extrapolated from disease behavior outside gestation. The principal determinants of adverse outcomes are active disease at conception, lupus nephritis (current or previous), antiphospholipid antibodies or antiphospholipid syndrome, chronic hypertension, accumulated organ damage, and anti-Ro/SSA or anti-La/SSB seropositivity. These factors do not confer identical risk throughout gestation; rather, they influence different maternal and placental pathways at different times in pregnancy (4,5,8,16,30).

During the first trimester, active systemic inflammation and antiphospholipid-mediated placental injury increase the risks of implantation failure, miscarriage and early pregnancy loss. In the second and third trimesters, placental malperfusion, endothelial dysfunction, nephritis and maternal vascular disease become increasingly relevant, translating into preeclampsia, fetal growth restriction, medically indicated prematurity, and stillbirth. Anti-Ro/SSA and anti-La/SSB antibodies introduce a distinct fetal risk profile, especially congenital heart block and neonatal lupus, which emerges during the mid-trimester period of greatest transplacental antibody effect (5,8,31-33).

Evidence-based counseling should therefore emphasize timing and stratification. Women who conceive in remission or low disease activity on pregnancy-compatible therapy usually have substantially improved outcomes than those who conceive during active disease or while receiving teratogens. Recent data confirm that active disease, nephritis, and antiphospholipid antibodies are among the most consistent predictors of adverse pregnancy outcomes, while pregnancy planning and medical readiness remain modifiable levers that clinicians can intervene upon before conception (4,17,23,30). This risk pattern is supported by systematic evidence identifying lupus nephritis, antiphospholipid antibody positivity, and active disease as recurrent predictors of fetal loss, preterm birth, hypertensive disease, and fetal growth restriction (4,30).

## 9. Maternal complications associated with SLE

### Preeclampsia

Preeclampsia is one of the most critical maternal complications associated with pregnancy in patients with SLE as it is both more common and more difficult to diagnose than in the general obstetric population. The risk further increases in the presence of lupus nephritis, chronic hypertension, antiphospholipid antibodies and active systemic inflammation. Previous research has consistently identified nephritis and antiphospholipid positivity as major risk amplifiers, supporting the concept that preeclampsia in SLE often reflects overlapping endothelial, placental, inflammatory, and renal pathology rather than an isolated obstetric event (4,5,17,30,34,35). Systematic reviews and meta-analyses specifically support lupus nephritis and antiphospholipid antibody positivity as major predictors of hypertensive disease and other adverse pregnancy outcomes (4,36). Primary cohort and guideline-based evidence further supports the contribution of chronic hypertension, active inflammation and renal impairment to preeclampsia risk in SLE (5,17,30,34,35).

Diagnostic differentiation from lupus nephritis flare is a major clinical challenge as both conditions may present with hypertension, proteinuria, thrombocytopenia and renal dysfunction. Features favoring nephritis include active urinary sediment, rising anti-dsDNA titers, hypocomplementemia relative to baseline, and concomitant extra-renal lupus manifestations. Features favoring preeclampsia include abrupt-onset hypertension following mid-gestation, placental insufficiency and angiogenic imbalance. Emerging evidence suggests that angiogenic biomarkers, particularly soluble fms-like tyrosine kinase-1 (sFlt-1), placental growth factor (PlGF) and the sFlt-1/PlGF ratio may serve as useful adjuncts in distinguishing preeclampsia from active lupus nephritis, although they should be interpreted within the broader clinical context rather than used in isolation (29,36).

From a preventive standpoint, low-dose aspirin should be initiated in women with SLE unless contraindicated, ideally commencing by 12-16 weeks of gestation, and anticoagulation should be added when obstetric or thrombotic APS is present (8,16,37).

### Hemolysis with elevated liver tests and thrombocytopenia (HELLP)

HELLP syndrome represents a severe microangiopathic and endothelial variant within the hypertensive disease spectrum of pregnancy and may be particularly complex in women with SLE as thrombocytopenia, hemolysis, transaminitis and renal impairment can overlap with lupus flare, thrombotic microangiopathy, catastrophic APS and active nephritis. In women with lupus who are pregnant, HELLP should therefore trigger a structured differential diagnosis rather than reflex attribution to preeclampsia alone (8,16,38).

Current evidence supports prompt multidisciplinary assessment, serial maternal laboratory monitoring and fetal evaluation, with delivery remaining the definitive management once maternal or fetal instability is established. Complement dysregulation has been implicated in severe disease phenotypes, which is mechanistically relevant in SLE and antiphospholipid syndrome, although complement-targeted therapy remains investigational rather than standard care in this setting (38).

### Lupus nephritis

Lupus nephritis is among the strongest predictors of adverse maternal and fetal outcomes in pregnancy in patients with SLE. Its importance lies not only in the presence of renal disease itself, but also in the timing of activity, the degree of baseline chronic damage, the burden of proteinuria, the coexistence of hypertension and difficulty distinguishing renal flare from superimposed preeclampsia. Women with active or recent nephritis have higher rates of preeclampsia, preterm delivery, fetal growth restriction and pregnancy loss than women with quiescent non-renal disease (4,5,17,34,35,39).

The most favorable outcomes are observed when pregnancy occurs after at least 6 months of renal quiescence, with stable serum creatinine, controlled blood pressure and low proteinuria. During pregnancy, worsening proteinuria alone is insufficient to diagnose nephritis flare as gestational physiology and preeclampsia can both alter urinary findings. Interpretation should instead integrate urinary sediment, complement trends, anti-dsDNA activity, blood pressure trajectory, extrarenal manifestations and fetal/placental assessment. Angiogenic biomarkers, particularly the sFlt-1/PlGF ratio, may add discriminatory value when clinical uncertainty persists (29,36).

Management depends on severity and timing. Hydroxychloroquine should be maintained; glucocorticoids are used for flares; and pregnancy-compatible steroid-sparing agents, such as azathioprine or calcineurin inhibitors may be required. Renal biopsy during pregnancy is reserved for selected situations in which the result would meaningfully alter management and procedural risk is acceptable. More commonly, clinicians rely on integrated clinical judgment and empiric treatment when the pretest probability of active nephritis is high (8,16,24).

### Obstetric APS and antiphospholipid antibodies

Obstetric APS is a major modifier of pregnancy risk in SLE and should be discussed not as an ancillary antibody finding, but as a distinct placental-thrombotic phenotype. Persistent lupus anticoagulant, anticardiolipin and anti-beta2 glycoprotein I antibodies are associated with recurrent early loss, fetal death, placental insufficiency, severe preeclampsia and prematurity. Among these markers, lupus anticoagulant remains the strongest single laboratory predictor of poor pregnancy outcomes (30,37,40).

Risk stratification should distinguish asymptomatic antibody positivity from definite obstetric APS and thrombotic APS, as management differs substantially across these categories. Women with prior obstetric APS generally require low-dose aspirin plus prophylactic heparin, whereas women with thrombotic APS usually require therapeutic anticoagulation throughout pregnancy and the postpartum period. Persistently positive antibodies without prior events warrant individualized counseling and, in numerous centers, low-dose aspirin-based prevention depending on the antibody profile and overall risk context (8,16,30,37).

### Sepsis

Infection deserves greater emphasis in pregnancy in patients with SLE as it contributes materially to maternal morbidity and may mimic flare, particularly when fever, cytopenia, elevated inflammatory markers, or multiorgan dysfunction are present. Pregnancy-related immune modulation, chronic corticosteroid exposure, baseline immune dysregulation, nephritis and the use of immunosuppressive therapy collectively increase susceptibility to severe infection. Clinically, the challenge lies in discriminating infection from active SLE, while avoiding delayed antimicrobial therapy in unstable patients (5,7,16).

### Hematological complications

Hematological complications in pregnancy in patients with SLE extend beyond descriptive anemia and thrombocytopenia. These abnormalities may reflect disease activity, medication toxicity, iron deficiency, microangiopathy, hemolysis, APS, or gestational physiology, and their prognostic significance depends on context. Persistent thrombocytopenia, hemolysis, or pancytopenia should prompt evaluation for flare, HELLP syndrome, thrombotic microangiopathy, or infection rather than being attributed automatically to uncomplicated gestation (5,8,16).

## 10. Placental disease in pregnancy in women with SLE and fetal complications of SLE

### Placental disease

Placental disease is central to the pathophysiology of adverse obstetric outcomes in SLE. Rather than being viewed as a passive end-organ victim, the placenta functions as a biologically active interface where interferon signaling, complement activation, antiphospholipid-mediated thrombosis, endothelial injury and maladaptive spiral artery remodeling converge. Histopathological analyses describe infarction, decidual vasculopathy, fibrin deposition and impaired villous development, all of which are compatible with chronic uteroplacental malperfusion (8,14,29,41).

This placental perspective helps explain why women with even mildly active systemic disease may still experience fetal growth restriction, preeclampsia and prematurity, particularly when serologic activity or antiphospholipid antibodies are present. It also underscores why fetal surveillance should be interpreted in parallel with maternal biomarkers and blood pressure rather than as a purely fetal issue.

### Fetal complications of SLE

Fetal complications of SLE should be framed according to the mechanism and timing. Early losses are more closely linked to antiphospholipid-mediated implantation and placental failure, whereas later complications are more often driven by placental insufficiency, maternal vascular disease, nephritis, hypertension and medically indicated preterm delivery. Across recent syntheses, the most reproducible predictors of poor fetal outcomes are active maternal disease, lupus nephritis, antiphospholipid antibodies, chronic hypertension and prior adverse pregnancy history (4,5,8,17,30).

Clinically, surveillance should be risk-adapted. Serial fetal growth assessment, umbilical artery Doppler when placental insufficiency is suspected, maternal blood pressure monitoring, and targeted fetal echocardiography in anti-Ro/SSA- or anti-La/SSB-positive pregnancies improve practical utility more than broad descriptive statements about risk alone (8,16,21).

*Neonatal lupus*. Neonatal lupus is a passively acquired autoimmune syndrome caused primarily by transplacental transfer of maternal anti-Ro/SSA and anti-La/SSB antibodies, with cardiac disease representing its most severe manifestation. Cutaneous lesions, cytopenia, hepatobiliary abnormalities and transient laboratory disturbances are often self-limited as maternal antibodies clear. By contrast, congenital heart block is usually irreversible and is associated with substantial morbidity, frequently requiring pacemaker placement (31-33,42,43).

As risk is antibody-mediated rather than disease-activity-mediated, even asymptomatic mothers with anti-Ro/SSA or anti-La/SSB positivity require structured surveillance. Current practice generally includes serial fetal echocardiographic assessment during the period of highest risk for conduction abnormalities, typically from ~16 to 26 weeks of gestation, with some centers extending surveillance thereafter in selected high-risk pregnancies. Hydroxychloroquine continuation is particularly critical, as prospective data support a reduction in recurrent congenital heart block among anti-SSA/Ro-positive pregnancies exposed to hydroxychloroquine (33). Thus, neonatal lupus is not merely defined entity, but as a condition requiring anticipatory screening, counseling regarding recurrence risk and coordinated fetal-cardiology follow-up. This recommendation is supported by reviews of neonatal lupus pathogenesis and clinical cohorts demonstrating the association between maternal anti-Ro/SSA or anti-La/SSB antibodies and fetal conduction disease (31-33,42,43).

## 11. Puerperium

The puerperium is a high-risk transition period in SLE. Hemodynamic shifts, postpartum inflammatory rebound, medication changes, interrupted follow-up and the risk of thrombosis, particularly in women with antiphospholipid antibodies all contribute to maternal vulnerability. Postpartum care should therefore be prespecified prior to delivery and include thromboprophylaxis duration when indicated, blood pressure follow-up, renal reassessment in women with nephritis or preeclampsia, medication reconciliation for lactation, and early rheumatology review as flares may emerge in the weeks to months following delivery (8,16,44,45).

## 12. Medications for SLE during pregnancy

Pharmacological management for SLE during pregnancy should be presented according to therapeutic intent and reproductive safety rather than as a list of isolated agents. The central objective is to maintain maternal disease quiescence using pregnancy-compatible therapy as uncontrolled lupus is consistently more dangerous to both the mother and fetus than the majority of approved medications used judiciously during gestation. Accordingly, therapy should be stratified into: i) Medications recommended to be continued; ii) medications that can be used when clinically indicated; iii) medications that require discontinuation prior to conception due to known teratogenicity or insufficient safety data (8,9,15).

### Corticosteroids

Glucocorticoids remain essential for the treatment of lupus flares in pregnancy, and their role should be described with greater therapeutic nuance. Non-fluorinated glucocorticoids such as prednisone and prednisolone are preferred for maternal disease as placental metabolism limits fetal exposure. They are effective for arthritis, serositis, cutaneous disease and a number of moderate flares; however, their prolonged or high-dose use increases the risks of gestational diabetes, hypertension, infection, preterm birth, osteoporosis, and possibly, growth restriction; thus, the lowest effective dose should be used and steroid-sparing therapy should be introduced when repeated escalation is anticipated (8,16,46,47).

Fluorinated corticosteroids, such as dexamethasone and betamethasone, cross the placenta more readily and are therefore reserved for specific fetal indications rather than routine maternal control. This distinction is particularly relevant in the context of suspected fetal conduction disease, where fluorinated steroids may be considered in selected early or incomplete block scenarios after multidisciplinary discussion, despite ongoing uncertainty in the evidence base (15,33,48).

### Non-steroidal anti-inflammatory drugs (NSAIDs)

NSAIDs may still play a role in the short-term control of pain and serositis in selected patients, although their use during pregnancy needs to be restricted by gestational age and maternal-fetal context. They are generally avoided in the third trimester due to the risk of ductus arteriosus constriction and oligohydramnios, and caution is also warranted from 20 weeks onward in light of fetal renal concerns highlighted by regulatory agencies. Consequently, NSAIDs should not be portrayed as routine background therapy in lupus pregnancy; instead, they are limited adjuncts, while acetaminophen and disease-directed anti-inflammatory control are often safer long-term strategies (6,16,49).

### Antithrombotic therapy

Antithrombotic therapy in pregnancy in patients with SLE is primarily determined by antiphospholipid antibody status, prior thrombosis and obstetric history. Low-dose aspirin is recommended for the majority of pregnant women with SLE to reduce the risk of preeclampsia, while heparin-based regimens are added according to the APS phenotype. Warfarin is generally avoided during organogenesis due to teratogenicity, although individualized exceptions in highly selected mechanical-valve or extreme-thrombotic-risk scenarios belong to specialist management rather than routine lupus pregnancy care (8,16,37,50,51).

### Immunomodulators

Hydroxychloroquine is the cornerstone maintenance therapy used in pregnant patients with SLE and should, in the majority of cases, be continued prior to conception, throughout gestation and during lactation. It is associated with lower disease activity and may reduce selected obstetric and neonatal complications, including the recurrence of congenital heart block in anti-SSA/Ro-positive pregnancies (6,6,33,52-54). For this reason, drug discontinuation out of unfounded teratogenic concern should be actively discouraged.

Azathioprine is among the preferred steroid-sparing immunosuppressants in pregnancy when additional disease control is needed, particularly for nephritis maintenance or persistent systemic activity. Its use should be limited to accepted dosing thresholds, although it is far better supported in pregnancy than several alternative immunosuppressants (8,16,55,56).

### Immunosuppressants

Mycophenolate mofetil, mycophenolic acid, methotrexate and cyclophosphamide are contraindicated or strongly avoided in pregnancy due to established embryotoxicity, fetotoxicity, or gonadotoxicity. Their importance lies not only in identifying them as unsafe agents, but also in emphasizing preconception transition planning. Women receiving these therapies require early counseling, reliable contraception and deliberate conversion to pregnancy-compatible alternatives before conception is attempted (8,16,57-59).

Calcineurin inhibitors on the other hand, such as tacrolimus and cyclosporine, are increasingly recognized as useful pregnancy-compatible options for selected patients with lupus nephritis or steroid-dependent disease, whereas voclosporin, despite efficacy in active lupus nephritis outside pregnancy, lacks sufficient pregnancy safety data to recommend its use during gestation. The appropriate framework is therefore: Established calcineurin inhibitors may be considered in pregnancy when indicated, and voclosporin is relevant primarily to pre-pregnancy risk assessment and drug transition planning (24,60).

## 13. Biologics and emerging therapies

This section briefly discusses biologics and emerging therapies to reflect current SLE therapeutics. Anifrolumab is currently an approved treatment for moderate-to-severe SLE; some women of reproductive age may present for preconception counseling while receiving it. However, human pregnancy data remain insufficient to recommend routine continuation during gestation; thus, current practice generally favors transition to better-characterized pregnancy-compatible regimens prior to conception whenever disease control allows (61). Belimumab has a growing, yet still incomplete pregnancy safety dataset and may be considered case by case in refractory disease, whereas rituximab is usually reserved for severe maternal indications in exceptional circumstances (9,16). Optimizing pharmacological management in SLE during pregnancy requires balancing maternal disease control with fetal safety through the use of pregnancy-compatible therapies and the avoidance of teratogenic agents (Table III).

Emerging classes such as Janus kinase inhibitors should also be mentioned in the context of reproductive counseling. Although these agents are not standard pregnancy therapies in SLE, they are increasingly encountered in immune-mediated disease management and are generally avoided in pregnancy due to limited human safety evidence. Their relevance to the present review lies in pre-pregnancy medication reconciliation, washout planning, and risk communication rather than use during gestation itself. This distinction improves the completeness and contemporary relevance of the pharmacologic discussion without overstating pregnancy safety data.

## 14. Conclusion

Pregnancy in women with SLE currently has a substantially improved prognosis than in earlier decades; however, favorable outcomes depend on one principle above all others: Conception should occur during sustained remission or low disease activity on pregnancy-compatible therapy. Across the current evidence base, the most consistent drivers of adverse outcome are active disease at conception, lupus nephritis, antiphospholipid antibodies/APS, chronic organ damage and inadequate pre-pregnancy optimization. Conversely, hydroxychloroquine continuation, aspirin prophylaxis when appropriate, anticoagulation for APS, and structured multidisciplinary surveillance meaningfully improve care (4-6,8,16,17).

Clinically, pregnancy in patients with SLE should be managed as a longitudinal pathway rather than an isolated gestational episode. This pathway includes reproductive planning and contraception during unsafe periods, rigorous pre-pregnancy assessment, trimester-specific maternal-fetal monitoring, prompt differentiation of flare from obstetric mimics such as preeclampsia, and postpartum follow-up for thrombosis, renal deterioration, hypertension, flares and medication-lactation reconciliation. Remaining knowledge gaps include the optimal role of biomarkers for nephritis vs. preeclampsia discrimination, long-term outcomes following *in utero* exposure to novel biologics and targeted therapies, and the refinement of individualized fetal surveillance strategies in antibody-positive pregnancies. Future research is warranted to focus on these clinically unresolved areas to support more precise and safer reproductive care for women with SLE.

## Figures and Tables

**Table I:** Preconception assessment framework for pregnancy planning in women with systemic lupus erythematosus.

Domain	Preconception assessment	Clinical rationale
Disease activity	Confirm remission or low disease activity for at least 6 months; document recent flare history and current symptom burden.	Active disease at conception is strongly associated with maternal flare, preeclampsia, prematurity and fetal loss.
Renal status	Serum creatinine, urine protein quantification, urinary sediment, blood pressure, and prior lupus nephritis phenotype.	Defines baseline maternal risk and improves later differentiation between nephritis flare and preeclampsia.
Antibody profile	aPL profile, anti-Ro/SSA, anti-La/SSB, anti-dsDNA, complement baseline.	Guides anticoagulation strategy, fetal echocardiographic surveillance, and inter-pretation of serologic change during pregnancy.
Medication plan	Continue pregnancy-compatible therapy; discontinue or switch teratogens with documented washout and replacement plan.	Prevents avoidable flare while minimizing embryotoxic or fetotoxic exposure.
Organ damage/comorbidity	Pulmonary hypertension, chronic kidney disease, heart failure, chronic hypertension, prior thrombosis or stroke.	Identifies pregnancies that should be delayed, co-managed intensively, or occasionally discouraged.
Obstetric history	Previous miscarriage, fetal growth restriction, preeclampsia, preterm birth, congenital heart block.	Determines recurrence risk and the intensity of maternal-fetal surveillance.

**Table II:** Recommended pre-pregnancy analyses and their pregnancy-specific clinical utility in women with systemic lupus erythematosus.

Analysis	Pregnancy-specific use
Complete blood count, creatinine, liver profile	Establishes maternal baseline and identifies cytopenia, renal impairment, or comorbidity relevant to pregnancy risk.
Urinalysis + protein quantification	Essential for documenting baseline renal involvement and later interpreting new proteinuria.
Anti-dsDNA, C3, C4	Best interpreted longitudinally; falling complement relative to baseline and rising anti-dsDNA may support flare.
Antiphospholipid antibody panel	Determines antiphospholipid syndrome risk stratification and aspirin/heparin planning.
Anti-Ro/SSA and anti-La/SSB	Identifies pregnancies requiring counseling about neonatal lupus and serial fetal rhythm surveillance.
Targeted echocardiography/pulmonary evaluation	Reserved for suspected cardiopulmonary disease, pulmonary hypertension, or prior organ involvement.
Medication reconciliation	Ensures compatible treatment, washout of teratogens, and clear pregnancy/lactation planning.

**Table III:** Medication use in pregnancy for women with systemic lupus erythematosus: Compatibility, restrictions and clinical considerations.

Medication/class	Pregnancy	Comment
Hydroxychloroquine	Continue	Cornerstone background therapy; associated with lower lupus activity and no established teratogenic signal.
Prednisone/prednisolone	Use if needed	Prefer lowest effective dose; useful for flare control, but prolonged high-dose use increases maternal complications.
Azathioprine	Compatible	Preferred steroid-sparing option when additional control is required.
Tacrolimus/cyclosporine	Compatible in selected cases	Useful particularly in nephritis or steroid-dependent disease; requires blood pressure and renal monitoring.
Low-dose aspirin	Recommended in most pregnancies	Used to reduce preeclampsia risk unless contraindicated.
Heparin	Use according to antiphospholipid syndrome phenotype	Preferred anticoagulant in pregnancy.
Non-steroidal anti-inflammatory drugs	Limited/avoid in late pregnancy	Short-term only in selected situations; avoid in the third trimester and use caution from 20 weeks onward.
Mycophenolate, methotrexate, leflunomide	Contraindicated	Require discontinuation before conception.
Cyclophosphamide	Avoid/contraindicated except in exceptional circumstances	Embryotoxic and gonadotoxic; relevant mainly to preconception planning.
Voclosporin	Insufficient pregnancy data	Important for preconception medication review; not recommended routinely in pregnancy.
Anifrolumab/JAK inhibitors	Insufficient pregnancy data	Should generally be discontinued or switched before conception whenever feasible.

## Data Availability

Not applicable.
